# Explaining site-level fidelity within a national initiative to implement a VA patient safety guidebook: the difference-making role of networks & communications

**DOI:** 10.1186/s43058-025-00797-8

**Published:** 2025-11-27

**Authors:** Jennifer L. Sullivan, Edward J. Miech, Marlena H. Shin, Jeffrey A. Chan, Michael Shwartz, Ann Borzecki, Hassen Abdulkerim, Edward Yackel, Sachin Yende, Amy K. Rosen

**Affiliations:** 1 Transformative Health Systems Research to Improve Veteran Equity and Independence (THRIVE) Center of Innovation (THRIVE COIN), VA Providence Healthcare System, Capt. Jonathan H. Harwood Jr. Center for Research, Providence, Rhode Island USA; 2 School of Public Health, Department of Health Services, Policy and Practice, Brown University, Providence, Rhode Island USA; 3 Center for Healthcare Optimization and Implementation Research, VA Boston Healthcare System, Boston, MA USA; 4 Veterans Affairs Center for Health Information & Communication, Roudebush VA Medical Center, Indianapolis, IN USA; 5 Center for Healthcare Organization and Implementation Research, VA Bedford Healthcare System, Bedford, MA USA; 6 Department of Health Law, Policy and Management, Boston University School of Public Health, Boston, MA USA; 7 Section of General Internal Medicine, Boston University School of Medicine, Boston, MA USA; 8 VA National Center for Patient Safety, Ann Arbor, MI USA; 9 Veterans Health Administration Office of Integrated Veteran Care, Pittsburgh, PA USA; 10 Department of Surgery, Boston University Chobanian and Avedisian School of Medicine, Boston, MA USA

**Keywords:** Implementation fidelity, Contextual determinants, Patient safety

## Abstract

**Background:**

Implementation fidelity—the degree to which an intervention is executed as intended—is critical for evaluating healthcare interventions' success. Contextual determinants such as organizational culture, communication, and leadership influence how interventions unfold at the site level. The Veterans Health Administration (VA) developed the *Patient Safety Events in Community Care: Reporting, Investigation, and Improvement Guidebook* to improve standardization of patient safety reporting across VA-delivered and VA-purchased care. While the *Guidebook* aims to enhance reporting fidelity, little is known about which local contextual factors influence its implementation success across diverse VA sites. This study examined the contextual determinants associated with site-level variation in *Guidebook* implementation fidelity.

**Methods:**

We conducted a cross-sectional, mixed-methods evaluation of 18 geographically diverse VA Medical Centers. Data were collected from 32 interviews with 45 key personnel involved in Guidebook implementation. Using the 2009 Consolidated Framework for Implementation Research (CFIR), 12 constructs were rated at each site. Fidelity was assessed across three safety processes (reporting, investigation, and improvement) on a three-point scale. We used Coincidence Analysis, a configurational comparative method, to identify combinations of CFIR constructs (difference-makers) that consistently distinguished higher- from lower-fidelity sites.

**Results:**

*Guidebook* fidelity varied across sites (range = 0.23 to 1.59). We identified three key CFIR constructs associated with higher fidelity: Networks & Communications, Relative Priority, and Leadership Engagement. Of these, Networks & Communications was both a necessary and sufficient condition for higher fidelity, serving as a precondition for high levels of Leadership Engagement. Sites that rated highly in Relative Priority were more likely to fully implement *Guidebook* processes. These constructs fostered strong collaboration, timely information exchange, and internal alignment on the importance of patient safety reporting.

**Conclusions:**

Effective communication networks and perceived priority of the intervention were central to high-fidelity implementation of the VA’s safety reporting *Guidebook*. These findings highlight critical levers for improving implementation fidelity in complex healthcare systems. Targeted strategies that strengthen communication and emphasize the value of safety interventions may enhance implementation success, offering valuable insights for patient safety efforts both within and beyond the VA.

Contributions to the literature

- We identify Networks & Communications and Relative Priority as key drivers of high-fidelity implementation.
- This project focuses on patient safety reporting where few implementation-related evaluation have previously focused.
- We employed a relatively new form of configurational analysis known as Coincidence Analysis to identify the difference-makers of higher versus lower fidelity.

## Background

Understanding how local contextual factors affect site-level implementation fidelity is essential for assessing healthcare interventions [1]. Implementation fidelity refers to how closely an intervention is carried out as intended, including adherence to core components and accurate delivery [2]. Contextual determinants—factors such as organizational culture, leadership, resources, and teamwork—can influence the implementation process and outcomes [2, 3]. Identifying these determinants can help uncover barriers and facilitators, enabling the development of targeted strategies to improve implementation and intervention effectiveness [4, 5].

Specifically related to our study, a review of 110 articles found that there were several factors affecting patient safety reporting (our focus of inquiry*)*. These include: organizational norms, work environment, team, process and systems of reporting, knowledge and skills, as well as staff and patient characteristics [6]. In addition to these multi-level factors, considerations regarding the incident itself and its mitigating factors (i.e., circumstances that moderate the progression of as incident toward patient harm) were also considered important in incident reporting [7]. For example, in a case study of 6 VA hospitals, sites with higher quality based on Patient Safety Indicators (PSIs), had higher levels of leadership support and better coordination of work and staff communication processes in place [8]. However, fewer studies to date have focused on factors affecting strategies to improve patient safety reporting, such as the use of a “guidebook” which typically outlines policies, procedures, and best practices to guide hospital staff in implementing patient safety initiatives designed to improve patient safety reporting. One recent study found barriers impacting guidebook roll-out to improve patient safety reporting including: planning for and executing guidebook implementation; engaging relevant staff in implementation; having available resources within the organization; networks and communications of staff; organizational culture; and external policies [8].

Although voluntary reporting of patient safety events has been a major component of the Veterans Health Administration’s (VA’s) patient safety initiatives [9], it has increasingly become more challenging to implement effectively, particularly with the challenges related to new legislative policies, such as the 2018 VA MISSION Act. This Act expanded options for Veterans to receive care in the community through VA-purchased care (Community Care) [10, 11]. The passage of the MISSION Act created a greater need for coordination of care and policies between VA and Community Care providers, as well as Third Party Administrators across the two care settings- VA-delivered care (VA) and Community Care. To address these challenges, the VA developed the "Patient Safety Events in Community Care: Reporting, Investigation, and Improvement Guidebook" (“*the Guidebook*”), aimed at standardizing and strengthening safety reporting between VA and Community Care [8]. The *Guidebook*promotes timely reporting, identifies system weaknesses, and supports continuous improvement through corrective actions. It is regularly updated to reflect new policies, such as the MISSION Act [10, 11].

While development and implementation of the *Guidebook* was an important step in improving standardization of patient safety reporting across VA care settings, research evaluating how successful the *Guidebook *was as a strategy in addressing its goals and challenges nationwide is limited [12]. Further, there is little understanding of the specific factors related to successful implementation of system-wide reporting of patient safety events in VA. To fill these gaps, this project examines the contextual determinants associated with variation in site-level fidelity within the national implementation of the *Guidebook *across all VA/Community Care settings. Fidelity assessment is used to understand the extent to which an intervention was implemented as intended and to better enable conclusions about its effectiveness in achieving targeted outcomes. We also use a novel analytical approach, Coincidence Analysis (CNA), to explore the complex interplay between local contextual factors and implementation fidelity [13]. CNA identifies “difference-makers” that uniquely and consistently distinguish sites with higher fidelity from those with lower fidelity in *Guidebook* implementation.

The VA is well-positioned to conduct this kind of evaluation as it is a large, integrated healthcare system with years of experience with national policy-driven initiatives. It also has a robust electronic health record infrastructure and a strong commitment to fostering a culture of patient safety and continuous improvement [14–16]. The insights gained from this evaluation have the potential to advance implementation science and improve patient safety practices across both the VA and Community Care settings.

## Methods

We conducted a cross-sectional, mixed-methods evaluation. We first analyzed qualitative data collected between February 2020 and January 2022 from interviews with key informants at 18 Veterans Affairs Medical Centers (VAMCs) that implemented the *Guidebook*. We then used quantitative methods to examine each individual site’s fidelity. Next, we applied CNA to identify key difference-makers (i.e., factors that consistently and uniquely distinguish sites with and without the outcome) explaining higher versus lower fidelity at the site level [17]. Findings from both the CNA and qualitative analyses were used to create a summary report, provide actionable insights for improving implementation fidelity across VA sites, and to inform our operational partners who helped develop and implement the *Guidebook*. The Institutional Review Board and Research and Development Committee at the VA Boston Healthcare System determined that this was a quality improvement project and was therefore exempt from Institutional Review Board oversight.

### Site selection and sample

Sites were selected based on geographic diversity, size (e.g., number of inpatient beds), and recommendations from operational partners as detailed in our previous work [8]. Participants included key informants such as Patient Safety Managers (PSMs), Quality Managers (QMs), and VA employees working on Community Care across the 18 VAMCs. These individuals were selected based on their knowledge about *Guidebook* implementation and their insights into the barriers and facilitators of the *Guidebook’s *processes [8].

### Prior data collection and analysis

We have previously published on our qualitative data collection and analysis procedures [8]. In this work, we used an interview guide informed by the *Guidebook’s *safety processes and constructs from the 2009 Consolidated Framework for Implementation Research (CFIR), a widely applied framework to guide the selection of factors that influence implementation of interventions [18]. Three health services researchers (MS, JS, JC) conducted 21 individual and 11 group interviews using Microsoft Teams™. Cross-site comparisons were organized in a matrix which identified 12 CFIR constructs that emerged as facilitators and barriers to implementation (MS, JS). This cross-site matrix was used to inform CFIR ratings below.

#### CFIR ratings

We used an aggregated cross-site matrix with summary evidence and supporting quotes from the 32 qualitative interviews to apply CFIR ratings. (See Table 1 for CFIR construct definitions which were based on original definitions) [18]. Using Damschroder and Lowery’s (2013) methods [19], constructs were rated based on both the valence and how strongly the determinant was present. Evidence to support the ratings included the amount and quality of the information from respondents who had the most experience with *Guidebook *implementation (i.e., PSMs, QMs, and VA employees working on Community Care). The rating scale was: −2 = At least two informants identified barriers, −1 = One informant identified a barrier, 0 = Mixed evidence from at least two informants, 1 = One informant identified a facilitator, or 2 = At least two informants identified facilitators [19]. One primary analyst (JS) reviewed the matrix and applied initial CFIR ratings based on the summary evidence and supporting quotes in the matrix. A second reviewer (MS) then reviewed the primary reviewer’s ratings, noting whether they agreed with the rating based on the information in the matrix including supporting evidence and quotes. To resolve any disagreements, the two raters met to come to 100% consensus on the final rating for each construct. Twelve CFIR constructs were scored using this method: Engaging, Executing, Planning, Reflecting & Evaluating, Available Resources, Culture, Implementation Climate, Leadership Engagement, Networks & Communications, Relative Priority, External Policies & Incentives, and Cosmopolitanism.

**Table 1:** Consolidated framework for implementation research construct definitions [16]

CFIR Construct	Definition
Engaging	Attracting and involving appropriate individuals in the implementation and use of the intervention through a combined strategy of social marketing, education, role modeling, training, and other similar activities
Executing	Carrying out or accomplishing the implementation according to plan
Planning	The degree to which a scheme or method of behavior and tasks for implementing an intervention are developed in advance, and the quality of those schemes or methods
Reflecting and Evaluating	Quantitative and qualitative feedback about the progress and quality of implementation accompanied with regular personal and team debriefing about progress and experience
Available Resources	The level of resources dedicated for implementation and on-going operations, including money, training, education, physical space, and time
Culture	The shared values, beliefs, and norms of an organization
Implementation Climate	Capacity for change, shared receptivity of involved individuals to an intervention, and the extent to which use of that intervention will be rewarded, supported, and expected within their organization
Leadership Engagement	Commitment, involvement, and accountability of leaders and managers with the implementation
Networks and Communication	The nature and quality of webs of social networks and the nature and quality of formal and informal communications within an organization
Relative Priority	Individuals’ shared perception of the importance of the implementation within the organization
External Policies and Incentives	Any external influence that can encourage or incentivize implementation of a new practice or program
Cosmopolitanism	The degree to which an organization is networked with other external organizations

#### Fidelity ratings

Fidelity was our primary outcome measure. It is defined as the degree to which an intervention is delivered as intended [2]. Each site’s implementation of safety processes (reporting, investigation, and quality/safety improvement) was rated on a three-point scale: 0 = No evidence of process implementation; 1 = Process partially implemented; 2 = Process fully implemented. A total of 13 elements, based on the key steps outlined in the *Guidebook,* were rated for reporting, investigation, and quality/safety improvement. Reporting consisted of 5 elements: 1) Event reported in JPSR; 2) Initial review of submitted patient safety event reports and completion of any immediate actions; 3) For high severity/probability patient safety events, notify appropriate personnel; 4) Scored event and determined action needs; 5) Include leadership in review process about. Investigation consisted of 5 elements: 1) Reviewed event and conduct initial event investigation for Community Care related patient safety events; 2) Complete root cause analysis facilitation training; 3) Participate on root cause analysis teams; 4) Perform/facilitate root cause analysis or aggregated reviews; 5) VA Patient Safety Officer and VA hospital community care coordination staff to provide support to VA hospital patient safety managers. Improvement consisted of 3 elements: 1) Participate in local patient safety/quality committee structure and share lessons learned and/or corrective actions across organization; 2) Facilitate gathering of patient safety data utilized for local trend analysis and provided patient safety metrics and reporting trends; 3) Conduct other improvement work, education, or peer review. To conduct the fidelity ratings, one primary analyst (MS) reviewed the summary evidence and supporting quotes in the matrix for each of the elements for reporting, investigation, and quality/safety improvement. Thereafter, another analyst (JS) conducted a secondary review of the primary analyst’s ratings along with the supporting summary evidence and quotes. When disagreements in ratings occurred, the two analysts met to discuss the information and reach consensus on the final fidelity ratings. Site-level scores for the individual safety processes of reporting, investigation and improvement were summed and averaged to generate an overall fidelity score for each site, distinguishing between higher- and lower-fidelity sites.

### Data integration

To integrate findings, fidelity scores were combined with CFIR ratings into a cross-site matrix, following Miles and Huberman’s analytical approaches [20]. This matrix enabled comparison across sites, identifying patterns influencing implementation fidelity.

#### Quantitative analysis

##### Coincidence analysis

After compiling the dataset consisting of the 12 CFIR ratings for each of the 18 different sites in the cross-site matrix, we (EM) next analysed the matrix using a relatively new form of configurational analysis known as Coincidence Analysis (CNA) to identify the “difference-makers” of higher versus lower fidelity [13, 21]. CNA identifies “difference-makers” that uniquely and consistently distinguish sites with and without an outcome of interest [22, 23]. CNA has been used in multiple prior studies to evaluate implementation of national VA programs [22–25].

The CNA algorithm is custom designed to manage complexity by modeling both conjuncts and disjuncts. Conjuncts refer to conditions that must all occur together to explain an outcome, using the Boolean operator "AND." For example, a hypothetical three-condition conjunct might be “factor A at the + 2 level,” “factor B at the + 2 level,” and “factor C NOT at the −2 level” must all be jointly present for the outcome to appear. Disjuncts, on the other hand, occur when different pathways lead to the same outcome, using the Boolean operator "OR." For instance, “Pathway 1” or “Pathway 2” could both lead to the same result, where each pathway is itself a conjunct of conditions. Another advantage of CNA is that its algorithm systematically identifies “difference-makers” through the application of Boolean algebra, set theory, and formal logic and thus can be used with datasets large or small, including small-n studies [13].

A three-step approach was used to conduct the CNA analyses: 1) factor calibration, 2) data reduction, and 3) model development [26]. Software used to support this analysis included the R package “cna” for Coincidence Analysis, RStudio, R, and Microsoft Excel (Ambuhl et al., 2022) [26, 27].

1. Factor calibration

Because our CNA analysis required categorical data, we dichotomized fidelity, the outcome factor for site-level implementation. Table 2 shows the distribution of mean fidelity scores across the 18 sites, which ranged from 0.23 (lowest) to 1.59 (highest), with a mean of 1.06. A large gap in the distribution of mean fidelity values occurred between 0.89 and 1.03 (i.e., sites 4 and 17 in Table 2), which also directly corresponded to the “process partially implemented” rating of “1” in the original scoring of the fidelity measure. Reviewing these data, the project team elected to assign a value of “1” (higher fidelity) to sites with mean fidelity scores ≥ 1, while those with scores < 1 were assigned “0” (lower fidelity). The 12 CFIR constructs, rated on a + 2 to −2 scale, were included as potential explanatory factors.

**Table 2:** Distribution of fidelity score averages across 18 sites

SITE	FIDELITY-SCORE-AVERAGE	OUTCOME_ FIDELITY
5	1.59	1
3	1.53	1
1	1.43	1
10	1.36	1
19	1.33	1
14	1.2	1
6	1.18	1
13	1.17	1
18	1.05	1
11	1.05	1
12	1.04	1
17	1.03	1
4	0.89	0
15	0.89	0
7	0.86	0
16	0.67	0
2	0.62	0
8	0.23	0

2) Data reduction

For data reduction, we used the "minimally sufficient conditions" (msc) function in the R “cna” package to analyze the 18 sites and 12 CFIR constructs. This function identified specific combinations of conditions that strongly related to the presence or absence of the fidelity outcome. We ran the msc function at five different consistency levels: 95%, 90%, 85%, 80%, and 75% [25, 26, 28]. From the results, we focused on configurations with the best coverage scores that were also consistent with logic, theory, and prior knowledge. This process allowed us to identify factors for model development in the next step.

3) Model development

The goal of model development was to create a reliable model with high consistency and adequate coverage. Consistency measures reliability, indicating how often cases identified by the model also exhibit the outcome, while coverage indicates how many cases with the outcome are included in the model. We aimed for a model with at least 90% consistency, 75% coverage, and no ambiguity (i.e., only having a single solution) [21].

## Results

### Site characteristics and participants

Geographic distribution and site size varied across 18 sites. At the 18 sites, we interviewed a total of 45 key informants [8]. Geographic distribution of the 18 sites varied, with the lowest number of sites from the Northeast region and the greatest number from the Midwest and West regions. The size of the 18 VAMCs also varied, ranging from 36 to 678 inpatient beds. We interviewed a total of 45 key informants: 19 PSMs, 7 QMs, and 19 CC staff at the 18 VAMCs. Additional site characteristics and the number of participants interviewed are available in Sullivan et al. 2024. [8]

### Coincidence analysis

Three of the twelve CFIR constructs played a key role in the fidelity outcome—Networks and Communications, Relative Advantage, and Leadership Engagement. The CFIR construct Networks & Communications played a key role in the fidelity outcome. (See Table 1 for construct definitions).

**Fig. 1:**
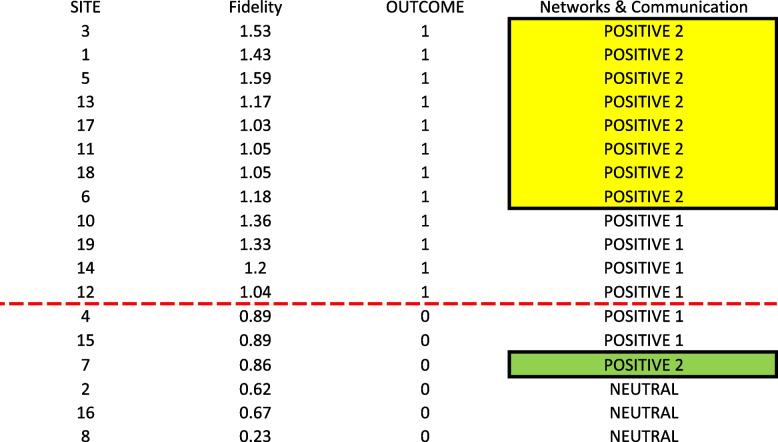
Visualization of the link between Networks and Communications at the Positive 2 level and sites with higher levels of implementation fidelity. Legend: The dotted red line separate the 12 cases with the outcome of higher fidelity (above the line) from those without (below line). Cells with yellow highlighter both have the outcome present and are covered by the model (i.e., consistent cases) whereas cells with green highlighter are covered by the model but do not have the outcome present (i.e., inconsistent cases)

Ratings levels for Networks & Communications were crucial. As shown in Fig. 1, a Positive 2 rating for Networks & Communication yielded the presence of the fidelity outcome (OUTCOME = 1), while a Neutral rating yielded the absence of the outcome (OUTCOME = 0). The Positive 2 and Neutral ratings were the highest and lowest observed ratings, respectively, for the Networks & Communications construct. A Positive 2 score for Networks & Communications was one reliable path to achieving the fidelity outcome, but not the only one. As shown in Fig. 1, sites 10, 12, 14, and 19 were not covered by this path, suggesting that other factors must also contribute to achieving the fidelity outcome.

**Fig. 2:**
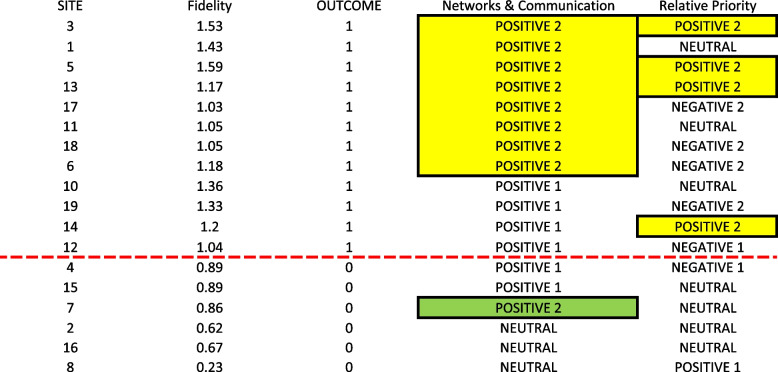
Final two- pathway high- reliability model accounting for sites with higher levels of implementation fidelity. Legend: The dotted red line separate the 12 cases with the outcome of higher fidelity (above the line) from those without (below the line). Cells with yellow highlighter both have the outcome present and are covered by the model (i.e., consistent cases) whereas cells with green highlighter are covered by the model but do not have the outcome present (i.e., inconsistent cases)

The second reliable pathway in the fidelity model was a Positive 2 rating for Relative Priority. As shown in Fig. 2, Relative Priority scores varied widely across the 18 sites, but a Positive 2 rating consistently linked to the outcome. This pathway uniquely explained Site 14, which did not have a Positive 2 rating for Networks & Communications. The high-reliability model resulting from the disjunction of the two pathways is shown in Fig. 2: Networks & Communications = POSITIVE 2 OR Relative Priority = POSITIVE 2 ↔ OUTCOME = 1. This model achieved a consistency score of 90% (9/10) and a coverage score of 75% (9/12). For example, Site 3 had higher levels of implementation fidelity due to a positive + 2 in both Networks and Communication and Relative Priority. For Networks and Communication, the quality manager in the VA community care department and patient safety manager closely worked together to ensure that the patient safety event reports were reviewed and any immediate actions were completed. In addition, they emphasized the importance of “closing the loop” with and providing feedback to staff who were involved in the reporting and investigating of events about the actions taken. For Relative Priority, leadership placed priority and importance on learning about and staying informed on when community care safety events occurred and thus staff placed priority on working through reporting, investigating, and improvement processes when these patient safety events were reported.

**Fig. 3:**
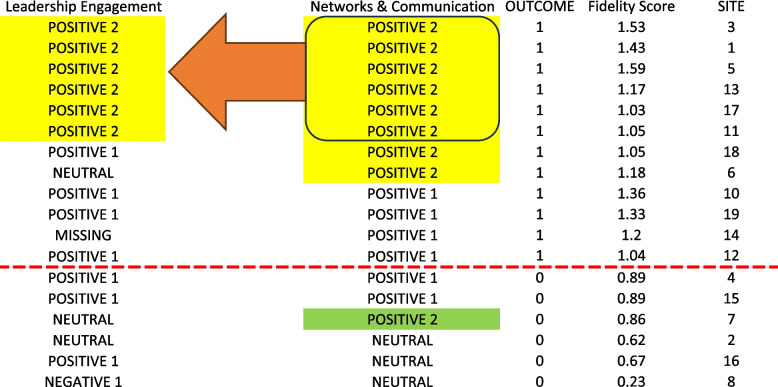
Visualization of Networks & Communications at the Positive 2 level as a necessary precondition for Leadership Engagement at the Positive 2 level

A supplemental finding relates to the interaction between CFIR constructs. As shown in Fig. 3, a Positive 2 rating for Networks & Communications was necessary, but not sufficient, for a Positive 2 rating for Leadership Engagement. This suggests that in this initiative, a Positive 2 score for Networks & Communications was a prerequisite for achieving a Positive 2 rating in Leadership Engagement, but not vice versa. In other words, while a Positive 2 rating for Networks & Communications did not automatically result in a Positive 2 rating for Leadership Engagement, sites could not achieve the highest level of Leadership Engagement without first reaching the highest level for Networks & Communications. At Site 5, the patient safety manager and staff in the VA community care department engage together in investigation process of community care related patient safety events by reviewing the report as well as obtaining information on and discussing with patients about what occurred. Thereafter, improvement action plans are developed. Leadership also is involved closely – this includes reviewing reports on patient safety events and trends seen as well as working with the VA community care department staff and patient safety manager on process improvements through the site’s Community Care Quality Oversight Council.

## Discussion

This project aimed to examine which contextual determinants explained site-level fidelity of the *Guidebook’s* implementation. We found variation in *Guidebook* fidelity, ranging from 0.23 (lowest) to 1.59 (highest), We identified two critical CFIR constructs at the Positive 2 level that linked directly to higher fidelity: Networks & Communications and Relative Priority. Moreover, Networks & Communications was the sole CFIR construct where the highest observed rating for that construct (i.e. Positive 2) consistently linked to the presence of the fidelity outcome and the lowest observed rating (i.e., Neutral) consistently linked to its absence. Additionally, we found that achieving a high score for Networks & Communications was a necessary precondition for obtaining a high score in Leadership Engagement. These findings highlight key factors influencing the successful implementation of the *Guidebook* at the local level and have broader implications for improving patient safety initiatives.

Our project reinforces the importance of effective communication networks and perceived relative priority in driving implementation fidelity. Networks & Communications emerged as both a facilitator and a necessary precondition for high fidelity. This construct fosters peer support, learning, and the effective exchange of information among key partners, ensuring alignment with *Guidebook *processes [29–33]. Similarly, Relative Priority reflects the perceived value and advantage of the intervention, motivating key partners to adopt *Guidebook *use. When these factors are optimized, they create a supportive environment with open communication at the site that is conducive to high fidelity in implementing patient safety processes [34–36]. The positive relationship between implementation outcomes and contextual determinants deserves further exploration. For example, high fidelity to the *Guidebook *may, in turn, strengthen communication networks and enhance perceptions of the intervention's value, creating a reinforcing cycle. Understanding and leveraging these dynamic interactions can inform strategies to sustain and scale implementation efforts across diverse healthcare settings [20, 37].

Our findings are aligned with previous work on patient safety reporting more generally which is aligned within the CFIR inner setting domains (i.e., networks and communications and relative priority) [6, 7]. While there is evidence linking *Guidebook* implementation to improvements in the patterns and quality of patient safety event reporting within VA—from both quantitative reporting trends and qualitative implementation studies [8–12], explicit causal analyses to isolate the *Guidebook’s* effect on reporting fidelity are limited. The existing evidence strongly suggests that the *Guidebook,* when supported by organizational infrastructure, contributes to enhanced reporting outcomes. Little research has been done testing implementation strategies–such as the *Guidebook*–within the VA. Our previous work found several CFIR determinants that included: *Planning* for and *Executing Guidebook* implementation; *Engaging* relevant staff in implementation; and having *Available Resources* within the organization. *External Policies*did not emerge as being related to fidelity [8]. Yet, this work did not compare these factors to the implementation outcome of fidelity. While our work focused on the hospital level, other incident-level factors could play a role in fidelity, and resulting patient safety outcomes including patient safety reporting details and any mitigating factors related to the report [7].

Our findings align with the broader research field which has also emphasized the role of strong communication and collaboration in achieving implementation fidelity [38]. Effective communication channels among developers, implementers, and supervisors ensure clarity of goals, alignment on processes, and shared learning, which collectively drive adherence to program guidelines [39]. Furthermore, collaboration and networking among implementers provides opportunities for peer mentoring, problem-solving, and mutual accountability, fostering a shared commitment to fidelity [32–46]. Relative Priority is similarly well-documented as a driver of implementation success. Key partners are more likely to invest effort and resources into an intervention when it is perceived as having clear advantages and high value. These perceptions can be amplified through communication networks that disseminate evidence of the intervention’s benefits and success stories [45, 46].

Our results are useful in identifying pragmatic solutions (i.e., implementation strategies) to help overcome barriers in future *Guidebook *implementation efforts [47]. Past work has identified several implementation strategies to overcome barriers [8]. Our findings focused on 3 CFIR determinants related to fidelity. The CNA results will allow us to better target Expert Recommendations for Implementing Change (ERIC) strategies in the future [4]. For example, addressing barriers of relative priority, networks and communication, and leadership engagement may require a bundled approach that draws from both *Develop Collaborator Interrelationships* and *Train and Educate Collaborators* ERIC strategy groupings [48]. Within the *Develop Collaborator Interrelationships* group [48], strategies such as identify and prepare champions, build a coalition, obtain formal commitments, and involve executive boards align with prior recommendations to use local consensus discussions, network weaving, and formal leadership engagement in order to strengthen communication channels and shared ownership. From the *Train and Educate Collaborators* grouping [48], strategies such as providing ongoing consultation, conducting educational meetings, and creating a learning collaborative, can complement earlier emphasis on audit and feedback. Further, tailoring messages to align with organizational priorities facilitates the perceived importance of reporting. Selecting the most relevant strategies and bundling them together can target interrelated CFIR barriers simultaneously—improving communication flow, increasing organizational priority, and fostering visible leadership support—to create the conditions for sustained and high fidelity.

This project has both strengths and limitations. A notable strength of this project is the novel application of Coincidence Analysis to evaluate implementation fidelity. Given the inherent interest within implementation science in better understanding the interplay of real-world complexities with implementation outcomes, our findings also underscore the fit between CNA as a method and the field of implemenation science. There have been over 20 relevant articles published in the literature since 2020 [49], including studies that used determinants frameworks and construct scoring of qualitative data [50–52]. CNA was highlighted in the Third Edition of the implemenation science guidebook *Dissemination and Implementation Research in Health: Translating Science to Practice* [47, 53]. The CNA approach allowed us to identify not only the individual constructs driving fidelity but also the specific “difference-makers” that were consistently linked with successful outcomes. CNA's ability to handle complexity and its compatibility with small sample sizes, as noted earlier, makes it particularly well-suited for exploratory analyses, such as these, in healthcare settings.

However, some limitations warrant consideration. We used mean scores when calibrating our three-level fidelity outcome factor. While it is not unusual in health services research to use means when reporting on Likert-style items, it required us to assume equal intervals between the three levels of none, partial and full. While use of CNA is innovative, there are also considerations when using this approach. It may be less useful when there is a limited diversity in the configurations. The assumption that all conditions have been measured and included can limit generalizability about sufficiency or necessity. Finally, it relies on crisp distinctions between the levels of each condition or variable requiring initial work assessing data characteristics before running the CNA.

The small number of observations per site restricted the breadth of insights, although we mitigated this by interviewing informants who were directly involved in the intervention and therefore knowledgeable about the implementation process. Additionally, third-party administrators (contractors managing the Community Care Networks) were unable to be interviewed due to contractual non-disclosure agreements, which may have limited the comprehensiveness of our findings. This type of information would have been valuable to operational partners collaborating with researchers on improving the quality of VA community care.

Although 3 CFIR constructs were “difference-makers” in this analysis, more work is needed to further elucidate whether additional CFIR constructs would also link directly to other outcomes, such as cost or clinical patient safety outcomes. While our results illustrate the importance of inner setting characteristics within each site, there may be additional lessons to be learned related to the local (or national) outer setting that did not emerge as part of this evaluation. Although CFIR was developed to encompass many determinants and created from a broad assessment of the literature, future work might also consider patient safety reporting-level information such as incident details and mitigating factors [6, 7].

## Conclusions

Our findings suggest some important implications for practice and areas for future research. Strengthening Networks & Communications, a key contextual factor affecting fidelity in this paper, would help to improve implementation effectiveness. This could involve organizing regular cross-disciplinary meetings, establishing collaborative coalitions, or leveraging digital platforms to facilitate real-time information sharing. To address challenges related to Relative Priority, engaging implementation champions who can advocate for the *Guidebook’s *benefits and securing leadership buy-in are key strategies. Future research should investigate the impact of these specific factors on clinical outcomes, such as the timeliness and quality of patient safety reporting and resolution. Additionally, exploring strategies to overcome barriers in other CFIR constructs, such as Leadership Support, could further improve implementation approaches. Integrating longitudinal assessments will also provide insights into how these contextual determinants and fidelity evolve over time and interact with emerging challenges [48, 49].

Furthermore, the findings of this project contribute significantly to both the enhancement of safety reporting and broader patient safety initiatives, both within and beyond the VA system. The VA developed and implemented the *Guidebook* to standardize safety reporting across all VA settings. By examining the influence of contextual determinants—particularly Networks & Communications and Relative Priority—the project provides critical insights into the factors that support successful implementation of patient safety processes. Such findings are valuable for improving safety reporting mechanisms, highlighting the need for strong communication networks in fostering accountability and transparency. Additionally, the project's implications may be applicable to patient safety outside the VA, as the identified constructs can inform strategies in other healthcare settings. By leveraging these insights, healthcare organizations can create environments where effective communication and the perceived value of interventions drive higher fidelity in safety practices, thus improving patient outcomes across diverse healthcare systems.

In conclusion, this project contributes to the growing body of evidence on contextual determinants influencing implementation fidelity in healthcare. By identifying the critical roles of Networks & Communications and Relative Priority, we offer a framework for enhancing the effectiveness and sustainability of the *Guidebook* processes. These findings not only inform strategies to strengthen patient safety initiatives within the VA but also provide valuable lessons for broader healthcare contexts aiming to implement complex interventions with high fidelity.

## Data Availability

The data analyzed during the current study are not publicly available because participant privacy could be compromised.

## Abbreviations

CFIR: Consolidated Framework for Implementation Research
CNA: Coincidence Analysis
MISSION: Maintaining Internal Systems and Strengthening Integrated Outside Networks Act of 2018
PSMs: Patient Safety Managers
QMs: Quality Managers
VA: Veterans Health Administration
VAMC: Veterans Affairs Medical Centers
